# Combination Training in Aging Individuals Modifies Functional Connectivity and Cognition, and Is Potentially Affected by Dopamine-Related Genes

**DOI:** 10.1371/journal.pone.0043901

**Published:** 2012-08-28

**Authors:** Valentina Pieramico, Roberto Esposito, Francesca Sensi, Franco Cilli, Dante Mantini, Peter A. Mattei, Valerio Frazzini, Domenico Ciavardelli, Valentina Gatta, Antonio Ferretti, Gian Luca Romani, Stefano L. Sensi

**Affiliations:** 1 Department of Neuroscience and Imaging, University “G. d’Annunzio” Chieti-Pescara, Chieti, Italy; 2 Molecular Neurology Unit, Center of Excellence on Aging, University “G. d’Annunzio”, Chieti-Pescara, Chieti, Italy; 3 Department of Neurosciences, Laboratory for Neuro- and Psychophysiology, Catholic University of Leuven, Leuven, Belgium; 4 Department of Biochemistry, School of Motor Sciences, Kore University of Enna, Enna, Italy; 5 Department of Psychological sciences, University “G. d’Annunzio” Chieti-Pescara, Chieti, Italy; 6 Functional Genomics Unit, Center of Excellence on Aging, University “G. d’Annunzio”, Chieti-Pescara, Chieti, Italy; 7 Departments of Neurology and Pharmacology, University of California Irvine, Irvine, California, United States of America; Yale University, United States of America

## Abstract

**Background:**

Aging is a major co-risk factor in many neurodegenerative diseases. Cognitive enrichment positively affects the structural plasticity of the aging brain. In this study, we evaluated effects of a set of structured multimodal activities (Combination Training; CT) on cognitive performances, functional connectivity, and cortical thickness of a group of healthy elderly individuals. CT lasted six months.

**Methodology:**

Neuropsychological and occupational performances were evaluated before and at the end of the training period. fMRI was used to assess effects of training on resting state network (RSN) functional connectivity using Independent Component Analysis (ICA). Effects on cortical thickness were also studied. Finally, we evaluated whether specific dopamine-related genes can affect the response to training.

**Principal Findings:**

Results of the study indicate that CT improves cognitive/occupational performances and reorganizes functional connectivity. Intriguingly, individuals responding to CT showed specific dopamine-related genotypes. Indeed, analysis of dopamine-related genes revealed that carriers of *DRD3 ser9gly* and *COMT Val158Met* polymorphisms had the greatest benefits from exposure to CT.

**Conclusions and Significance:**

Overall, our findings support the idea that exposure to a set of structured multimodal activities can be an effective strategy to counteract aging-related cognitive decline and also indicate that significant capability of functional and structural changes are maintained in the elderly.

## Introduction

“If we can at least prevent some of the normal age-related decline from happening, even if it doesn’t eliminate the risk – if it just reduces the risk of developing Alzheimer’s disease or makes the quality of life a little bit better – I think we’ve gone a long way” [1].

Brain plasticity is an intrinsic characteristic of the nervous system that allows a continuous remodelling of brain functions upon physio-pathological conditions [2]. Although normal aging is associated with morphological modifications and decline of cerebral functions, brain plasticity is preserved in elderly individuals [3]. A growing body of evidence supports the notion that cognitive enrichment and aerobic training induce a dynamic reorganization of higher cerebral functions, thereby helping in maintaining operational skills in the elderly [1], [4]-[6] and reducing the incidence of Alzheimer’s disease (AD) [7].

Functional magnetic resonance imaging (fMRI) is a useful tool to investigate modifications of functional connectivity that, at least in part, reflect changes in brain plasticity [8], [9]. Functional neuroimaging studies have highlighted that baseline neuronal activity is affected by multimodal stimuli [10], [11]. Neuronal activity in resting condition, evaluated with blood oxygen level dependent (BOLD) fMRI, is organized in multiple and highly specific functional anatomical networks (resting state networks, RSNs) [12]. These RSNs show fluctuations between 0.01 and 0.1 Hz and include sensory-motor, visual, auditory, attention, language, and default networks [13].

Many studies have investigated the Default mode network (DMN), a functional system that includes the Medial Prefrontal Cortex (MPFC), the Posterior Cingulate Cortex (PCC), the Inferior Parietal Lobule (IPL), and the hippocampus (Hp) [11]. This network is associated with reflective activity and self-referential mental processes [14] and is a powerful tool to explore brain functioning in different physio-pathological conditions [8], [14], [15]. Aging is associated with an overall decrease of DMN activation [16] especially within the MPFC and the PCC [17], changes that some authors consider prodromic signs of AD [18].

The capability of the brain to respond to exogenous and/or endogenous changes is also affected by the combined activity of several neurotransmitters, including the dopaminergic system which plays a critical role in cognition [19]. A large number of studies have highlighted the role of dopamine signalling dysfunction in neurological and psychiatric disorders [like, for instance, Parkinson’s disease and Attention Deficit Hyperactivity Disorder (ADHD)]; however, in the aging brain, little is known about system changes in terms of capability to modulate functional flexibility in response to environmental changes [18].

The presence of polymorphic variants of dopamine-related genes has been associated with a range of neuropsychological syndromes [20], but relatively few studies have investigated how dopamine polymorphic variants affect the response to cognitive enrichment [21].

In the present study, using neuropsychological, occupational, fMRI, and cortical thickness, measurements along with analysis of dopamine-related gene polymorphisms, we evaluated morpho-functional changes occurring in the brain of healthy elderly people who were stimulated for six months with a set of structured multimodal activities (Combination Training; CT). End points of the study were: 1) cumulative effects on the activity of different RSNs [13], 2) effects on specific cognitive functions, and 3) on performances of daily living (ADL) activities.

## Materials and Methods

### Study Population

The study protocol was approved by our institutional Ethics Committee. All procedures were conducted in accordance with the principles expressed in Helsinki Declaration. Thirty elderly adults (15 men; 15 women; age range 60–75 years) were enrolled. All participants underwent medical, psychiatric, and neurological examinations and had neither history nor signs of drug abuse, stroke, hypertension, diabetes, psychiatric or neurodegenerative conditions. All subjects signed an informed consent and were randomized in two groups: training group (7 men and 8 women) and control group (8 men and 7 women). Groups were matched for age and years of education (8–18). All subjects showed non-pathological scores in the Mini Mental State Examination (MMSE; i.e., ≥25). Subjects were also tested with clock drawing, phonemic verbal fluency (FAS), Trail Making Test (TMT), Frontal Assessment Battery (FAB), and prose memory (Babcock story) tests. Failure in one or more of these tests was a criterion for exclusion from the study. For occupational evaluations we administered the interest checklist OPHI-II and the Occupational Therapy Evaluation (OT-E). OT-E is a simplified version of the Assessment Motor and Process Skills (AMPS) [22] which evaluates performances in ADL activities related to motor and process skills.

### Statistical Analysis of Neuropsychological and Occupational Tests

Two factor ANOVA followed by Tukey HSD post hoc test was performed using the general linear model (GLM) approach which takes account of unequal sample size of the study groups. First aligned rank transformation (ART) of data was performed using the ARTweb program available at http://faculty.washington.edu/aimgroup/proj/art/artweb/. Group and time were the investigated factors. Statistical significance was set at p<0.05. Statistical analysis was performed using Statistica 6.0 (Statsoft, Tulsa, OK) software.

### Combination Training

CT was structured with a set of multimodal activities that included: cognitive, aerobic, and sensorial stimuli as well as fun-recreational activities (for a detailed description of these activities see Table 1 and the full protocol shown in Supporting Information: Text S1). The treated group followed CT for six months while the control group individuals were instructed to not modify their daily routine for the same time period. Subjects of both groups underwent neuropsychological examination and were exposed to the same fMRI protocols at the beginning of the training (T0) and six months later (T6).

**Table 1:** Combination Training.

	Cognitive training	Aerobic training	Exposure to musical stimulation^3^
**First three months**			
*Monday*	Crossword	Walking	Listening to music
*Tuesday*	Book reading		
*Wednesday*	Sudoku	ADL activity of choice	Listening to music
*Thursday*	Group discussions or fun-recreation project		
*Friday*	Word puzzles	Dancing	Listening to music
*Saturday^2^*	Fun-recreation project		
*Sunday*	Rest		
**Last three months^1^**			
*Monday*	Logical puzzle grids	Walking	Listening to music
*Tuesday*	Book Reading		
*Wednesday*	Sudoku	ADL activity of choice	Listening to music
*Thursday*	Group discussions or fun-recreation project		
*Friday*	Transfer	Dancing	Listening to music
*Saturday^2^*	Fun-recreation project		
*Sunday*	Rest		

***Activities employed in the CT program.*** CT is structured in three sections: brain training, aerobic training and exposure to musical stimulation, each of these domains includes different daily activity. The overall level of difficulty of all activities was increased after the first three months. The program also included a fun-recreation project with group activities performed every fourteen days.

Notes:

1)Activities of the last three months were set to be more difficult.

2)The fun-recreation project involved social activities which were conducted with alternating the rotation and sequence of the activities month by month. The activities were:

▪Card playing in the first 3 months.

▪Use of cognitive software in the last 3 months.

▪Exposure to a structured group discussion on topics encompassing weekly relevant political and cultural events as well as critique of the books read during the cognitive training.

▪Preparation of sets of cognitive activities for the group.

3)While performing the cognitive training activities subjects were invited to listen to a selected list of musical pieces that included music from Vivaldi, Chopin, Debussy, Mozart, Wagner, Queen, Elvis Presley, and The Rolling Stones. This musical compilation belongs to a branded CD called “Supermind”.

### fMRI Acquisition and Statistical Analysis

fMRI imaging was performed at rest acquiring BOLD data in four runs lasting four minutes each followed by high resolution T1 anatomical images. Subjects were asked to relax while fixating the center point of a grey-background screen projected via an LCD projector and viewed via a mirror placed above the subject’s head. Foam padding was employed to minimize involuntary head movement. BOLD functional imaging was performed with a Philips Achieva 3T Scanner (Philips Medical Systems, Best, The Netherlands) equipped with an eight channel dedicated head coil, using T2*-weighted echo planar imaging (EPI) free induction decay (FID) sequences applying the following parameters: TE 35 ms, matrix size 64×64, FOV 256 mm, in-plane voxel size 4×4 mm, flip angle 75°, slice thickness 4 mm and no gap. Functional volumes consisted of 30 trans-axial slices, acquired with a volume TR of 1671 ms. High resolution structural images were acquired at the end of four fMRI runs via a 3D MPRAGE sequence with the following parameters: sagittal, matrix 256×256, FOV 256 mm, slice thickness 1 mm, no gap, in-plane voxel size 1 mm×1 mm, flip angle 12°, TR = 9.7 ms and TE = 4 ms. Image data processing was carried out using Brain Voyager QX 1.9 (Brain Innovation, Maastricht, The Netherlands). Functional image time-series were first corrected for differences in slice acquisition time, de-trended, realigned with structural images and warped into standard Talairach anatomical space as previously described [13]. RSN maps were generated by means of independent component analysis (ICA), a data-driven method that decomposes BOLD time-series into a set of independent spatio-temporal patterns (independent components; ICs) [23]. First, a single subject ICA was performed, separately for each of the four runs, using a plugin extension of BrainVoyager QX based on the FastICA algorithm [24]. Thirty ICs were extracted for each data set and scaled to spatial z-score maps. Later on, ICA decompositions from the four within-subject repetitions per session were clustered using the self-organizing group-level ICA (sog-ICA) algorithm [25] implemented in BrainVoyager QX. The whole procedure yielded, for each subject, 30 clusters with four components each. These four components were averaged in order to obtain a set of 30 within-subject components for session. IC spatial maps were scaled to z-scores to allow comparisons across sessions and subjects. In each IC map, the z-score value that is associated to a given voxel reflects the weight of IC time course with respect to the relative measured BOLD data, thereby providing an indirect indication of functional connectivity [23]. After exclusion of artefactual ICs based on the IC-fingerprint method, we selected ICs showing the largest spatial correlation with RSN templates obtained in a previous resting-state study conducted on healthy volunteers [13]. This approach is in line with other resting-state studies on the DMN and DAN and assumes that there is a canonical spatial pattern that allows a reliable detection at the single-subject level using a template-matching procedure [26].

For statistical analysis, individually clustered fMRI datasets were divided into four different groups: trained/not trained and T0/T6. For each group, a random effect analysis was performed on RSN single-subject maps to define brain areas consistently found at the group level [p<0.001 corrected for multiple comparisons with false discovery rate, (FDR)]. A two-factor variance analysis (ANOVA) with repeated measures (scores of the different subjects) was then performed (p<0.05 uncorrected). For ANOVA analysis we considered two parameters: group (control and trained subjects) and time (T0–T6). We therefore assessed: 1) the effect of group regardless of time; 2) the effect of time regardless of group; and 3) the combined effect of time and group (interaction). Furthermore, we carried out an ANOVA post-hoc analysis, by calculating a paired t-test between single-subjects maps for the trained group at T0 and T6 (p<0.04 uncorrected).

Additional statistical analyses were performed only for brain regions showing a significant ANOVA interaction (p<0.05 uncorrected; for a detailed description of the statistically significant fMRI results see Supporting Information: Table S1). Single-subject ICA z-scores were calculated for each RSN node that showed significant modifications and comparisons ran using either a paired t-test, when analyzing results within the same group, or an unpaired t-test for comparisons between groups.

Network nodes showing significant modifications of connectivity between trained/untrained and T0/T6 were also evaluated to investigate possible correlations between functional connectivity data and expression of DRD3 [*ser9gly (Ser = A1* allele*; Gly = A2* allele)] polymorphisms. Additionally, we performed a statistical descriptive analysis on the same areas, highlighting the average and standard errors of z-score values for each group.

Spearman correlation analysis (95% confidence level), using Statistica 6.0 software, was performed in order to verify the association between neuropsychological/occupational and fMRI data in control and training groups, respectively. Correlations were evaluated by plotting regional fMRI data and neuropsychological/occupational scores at T0 and T6 for each group. This approach reveals significant correlations between changes in functional connectivity and neuropsychological/occupational findings that are dependent either on time or CT exposure. The analysis therefore indicates that if a correlation is found in the training group and not in the control that positive match should be ascribed to CT exposure and not to the T0–T6 time interval. The same correlation procedure was employed when plotting regional fMRI data and cortical thickness results.

### Cortical Thickness and Statistical Analysis

Cortical surfaces were reconstructed from the high resolution anatomical 3D–MPRAGE data sets of each subject using FreeSurfer version 5.1 (http://surfer.nmr.mgh.harvard.edu). The automated processing stream included white matter segmentation and tessellation to identify grey/white matter and grey matter/pial surface interfaces. An experienced neuroradiologist visually inspected segmentation results and inaccuracies were manually corrected prior to subsequent processing. Thus, the produced maps are not restricted to the voxel resolution of the original data and allow detection of sub-millimeter differences. Summary tables of the cortical parcellations (156 anatomical structures for each hemisphere) were used for further statistical analysis. After the scans from the two time points were processed, cross-sectionally, with the default FreeSurfer workflow, an unbiased template from both time points was created for each subject and processed cross-sectionally. This unbiased template reduces the random variation in the processing procedure and improves the strength and sensitivity of the overall longitudinal analysis. One subject from both groups was excluded from further analysis due to errors encountered in the automated process which could not be corrected manually. Vertex-based difference maps between the two time points were then created, re-sampled to a common map created from all included scans and smoothed with a Gaussian kernel (full-width of half-maximum of 15 mm). A two-way two time-point longitudinal analysis, F-test (unsigned), was performed to model vertex-wise difference maps with group as two separate planes (two levels of a single discrete factor) with one continuous factor as covariate (age). Areas included in this evaluation were limited to the DMN and DAN and therefore no statistical correction was applied.

As with fMRI data, brain areas showing significant changes of cortical thickness between trained/not trained and T0/T6 pairs were analyzed to investigate possible correlations between these changes and the expression of the DRD3 [*ser9gly (Ser = A1* allele*; Gly = A2* allele)] polymorphisms.

### Genotyping

All study subjects were informed about the procedure and gave informed consent for the collection of genomic DNA for genetic analysis. DNA was isolated using the Master-AMP Buccal Swab and DNA Extraction Kit (Epicentre Technologies) in accordance with the manufacturer’s protocol. *DRD1, DRD2, DRD3, DRD4,* and *COMT* gene polymorphisms were genotyped according to polymerase chain reaction-restriction fragment length polymorphism technique using forward and reverse primers and digestion enzymes as previously described [27]-[31]. After digestion, PCR products were resolved via 3% agarose gel electrophoresis. The *DRD5* promoter dinucleotide was amplified using a hot-start PCR protocol as described by Mill and colleagues [32]. Fluorescent amplification products were separated on an ABI 3100 Genetic Analyzer (PE Applied Biosystems) and analysed using Genotyper (PE Applied Biosystems) software. Dat1 PCR amplification of the region flanking the DAT1 40-bp VNTR was carried out as previously described [33]. PCR products were run on 3% agarose gel. A 50-bp DNA ladder was used to identify the various repeat alleles by size (Table 2).

**Table 2:** Genotyping.

Polymorphisms	dbSNP ID	Primers	Restriction enzymes	Digestion fragments (bp)
DRD1–48A/G	rs 686	5′-GGC TTT CTG GTG CCC AAG ACA GTG-3′5′-AGC ACA GAC CAG CGT GTT CCC CA-3′	DdeI	A: 146,42,217G: 146 259
DRD2-TAQ-IA	rs 1800497	5′-CCG TCG ACG GCT GGC CAA GTT GTC TA-3′5′-CCG TCG ACC CTT CCT GAG TGT CAT CA-3′	Taq I	A1∶310A2∶180,130
DRD3-Ser9Gly	rs 6280	5′-GCT CTA TCTCCA ACT CTC ACA-3′ 5′-AAG TCT ACT CAC CTC CAG GTA-3′	MscI	Ser: 304,111, 47Gly: 206, 111, 98,47
DRD4-521C/T	rs1800955	5′-ATG AGC TAG GCG TCG GCG G-3′5’-GCA TCG ACG CCA GCG CCA TCC TAC C -3′	FspI	C: 108, 176T: 284
DRD5 promoter (TC)n repeat	–	5′-FAM_ATC CAC CCA CCT CGG CCT CCC AAA-3′5′-ATG CAA GGT CTT TTC CTC ATA TTG- 3′	–	*TC12∶450TC13∶452
COMT-Val158Met	rs4680	5′- GGA GCT GGG GGC CTA CTG TG -3′5′- GGC CCT TTT TCC AGG TCT GAC A -3′	NlaIII	Val: 114,36,35 bpMet: 96,36,35,18 bp
DAT1 VNTR	–	5′- TGT GGT GTA GGG AAC GGC CTG AG -3′5′- CTT CCT GGA GGT CAC GGC TCA AGG -3′	–	9-r: 440 bp,10-r: 480 bp11-r: 520 bp

* allele repeat size.

***Analysis of dopamine polymorphisms.*** List of investigated dopamine polymorphisms and the employed primers, restriction enzymes, and digestion fragments.

## Results

### Memory and Occupational Performances

No subjects were excluded because they failed one of the screening tests or did not reach the minimum requirement for compliance. A detailed description of statistical analysis results can be seen in Supporting Information (Tables S2 and S3). Control and treated subjects were evaluated at the beginning (T0) and the end of a six month (T6) period for their MMSE scores [34] in order to assess the baseline cognitive status of both groups. No statistically significant differences in mean MMSE scores were found within and between the two groups at both T0 and T6 (Tables S3, S4).

**Figure 1:**
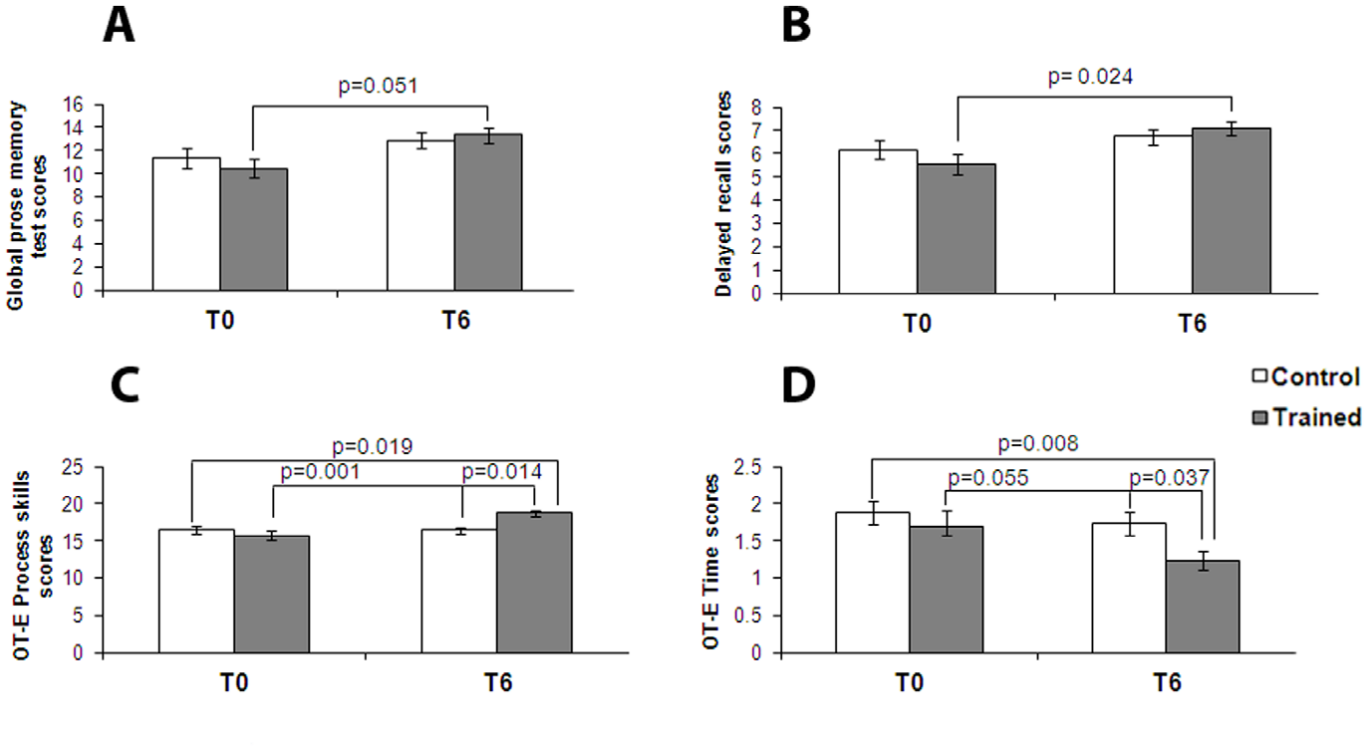
CT positively affects neuropsychological and occupational performances. Bar graphs depict results of neuropsychological evaluation in control (white bars) and trained (grey bars) groups at the beginning of the study (T0) and after six months (T6). Graphs show results, expressed as means ± SEM, of: (**A**) global prose memory test and (**B**) delayed recall subtest. The trained group shows, at T6, a statistically significant improvement in long-term memory compared to control individuals. In the bottom panel, graphs depict results, always expressed as mean ± SEM, of: (**C**) OT-E Process Skills and (**D**) OT-E Time. The training group shows, at T6, a statistically significant improvement in process skills compared to control group for the OT-E.

We also studied both groups for their performances in prose memory using the prose memory test (Babcock story) [35], a test that investigates short-term and long-term memory. Testing at T6 indicated that subjects treated with CT showed a positive trend that suggests improvement in prose memory test scores over the six month period (p = 0.051, Table S4) while the control group showed no changes over time (Fig. 1A). We further analyzed the scores of the prose memory test in the subset of tasks that are used to investigate immediate and delayed memory performance and found a significant increase in long-term memory in the treated group (p = 0.024, Table S4) while no changes were found in the control group (Fig. 1B). A statistically significant effect of time on prose memory test scores and delayed memory scores for both groups was found when results were analyzed by two factors ANOVA. The same analysis showed that control and trained subjects did not significantly differ for their neuropsychological baseline scores (Table S4).

We then studied the performance of both groups in their attention skills using TMT [36], a test that analyzes visual attention and task switching. When analyzing the results of different subsets, A and B (Test-A: sustained attention; Test B: divided attention; and Test B-A: task coordination and set-shifting) we found no statistically significant differences within and between the groups (Supporting Information, Tables S2, S3).

Analysis of FAB [37] and FAS [38], two tests that are employed to evaluate functioning of frontal lobes (FAB) as well as attention or lexical production (FAS) showed a global maintenance of these cognitive functions in both groups (Supporting Information, Tables S2, S3).

Finally, we investigated motor and process skills using OT-E [22]. ADL-related tasks chosen to evaluate motor skills (OT-E Motor Skills; a test that measures the ability to move and interact with tasks, objects, and environment), process skills (OT-E Process Skills; a test that assesses the ability employed in managing and modifying ADL actions), and reaction times (OT-E Time; a parameter evaluating the readiness/reaction times of the subject while performing ADL activities). OT-E Process Skills scores indicated that the trained group showed enhanced performance at T6 (p = 0.001, Supporting Information, Table S3) while the control group showed no changes (Fig. 1C). Furthermore, we found a significant interaction effect between group and time with OT-E Process scores significantly improved in the trained group over the investigated time. Analysis of OT-E Time scores revealed that the trained group showed a trend toward significance for improvement over the six month of training (p = 0.055, Supporting Information, Table S3) while the control group did not show any significant improvement (Fig. 1D).

### Brain Functional Connectivity

**Figure 2:**
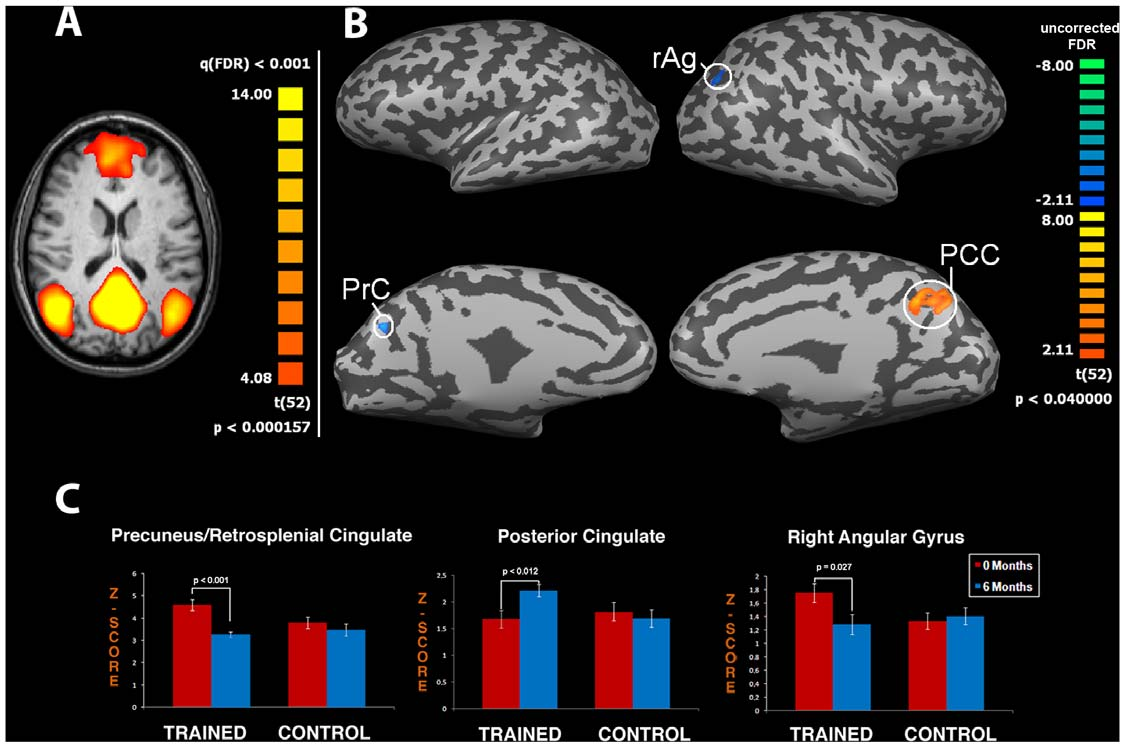
CT improves neuronal connectivity: effects on the Default Mode Network (DMN). Panel (**A**) depicts the DMN obtained from functional magnetic resonance (fMRI) data pooled from both groups [map threshold is t>4.08 and processed with independent component analysis (ICA)]. Panel (**B**) shows t-maps obtained by extrapolating the T6–T0 difference for the trained group. The procedure shows significant changes in the Precuneus (PrC, cluster size: 575 mm^3^), the Right Angular Gyrus (rAg, cluster size: 201 mm^3^), and the Posterior Cingulate Cortex (PCC, cluster size: 3081 mm^3^). Note that the trained group shows significantly increased activation in the PCC, an area that plays a crucial role in memory functioning. Functional maps are overlaid on a conventional inflated cortex with threshold of t>2.1. Graphs (**C**) show means ± SEM of z-scores of specific DMN areas (PrC, rAg and PCC).

**Figure 3:**
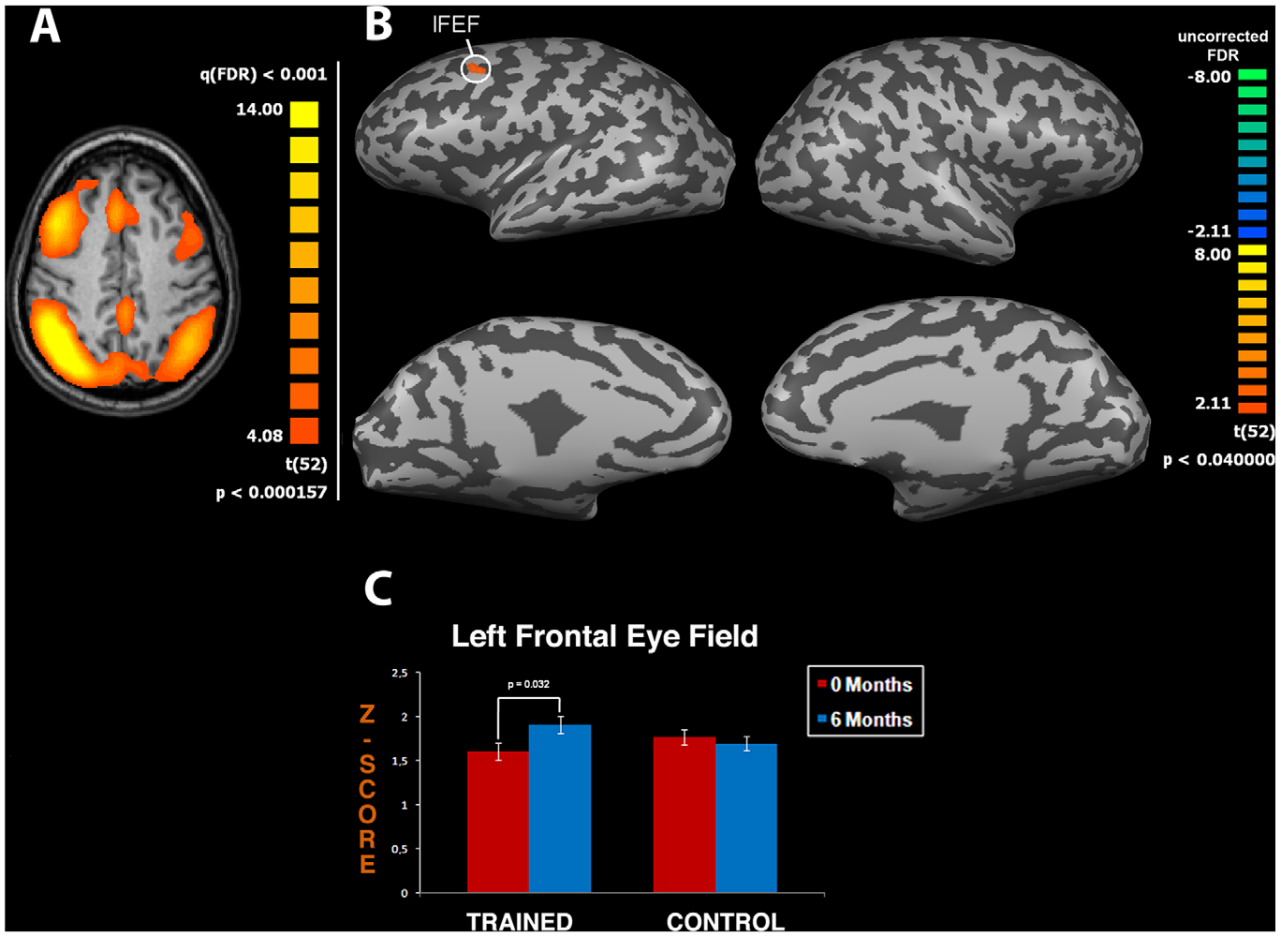
CT improves neuronal connectivity: effects on the Dorsal Attention Network (DAN). Panel (**A**) depicts the DAN obtained from functional magnetic resonance (fMRI) data pooled from both groups [map threshold is t>4.08 and processed with independent component analysis (ICA)]. Panel (**B**) shows t-maps obtained by extrapolating the T6-T0 difference for the trained group. The procedure shows significant changes in the Left Frontal Eye Field (lFEF, cluster size: 1381 mm^3^) an area that plays a critical role in the control of attention. Functional maps are overlaid on a conventional inflated cortex with threshold of t>2.1. Graph (**C**) shows means ± SEM of z-scores of lFEF.

FMRI analysis indicated that both groups showed typical DMN areas (p<0.001, FDR-corrected) as previously reported [13]. When we performed interaction analysis between T0 and T6 in both groups (ANOVA interaction, p<0.05 uncorrected) we discovered significant changes in connectivity of specific DMN and DAN areas. The same ANOVA analysis did not yield significant results if FDR was applied. Accordingly, these findings should be taken as exploratory and require further investigation in future studies employing larger cohorts of aging individuals. In the DMN we found changes in the strength of functional connectivity in the Precuneus (PrC), Right Angular Gyrus (rAg), and PCC regions in the trained group (t-test between T0 and T6, p<0.001, p = 0.027 and p<0.012, respectively; Fig. 2). Analysis of the DAN revealed a change in the strength of functional connectivity in the training group for the Left Frontal Eye Field area (lFEF; t-test between T0 and T6 in the training group, p = 0.032; Fig. 3). The cluster size of nodes showing significant changes is reported in the legend of Fig. 2 and 3. Analysis of remaining RSNs [13] did not show significant modifications.

ANOVA analysis of possible correlations between the investigated genotypes and connectivity findings did not reveal a significant facilitation exerted by specific polymorphisms on effects elicited by CT.

Finally, we performed a correlation analysis between neuropsychological/occupational results and fMRI data. For the trained group, we found a significant negative correlation between the strength of functional connectivity of PrC, delayed recall (R = −0.430, p = 0.036), and OT-E process skills (R = −0.546, p = 0.006) scores. Negative correlations between rAg and delayed recall (R = −0.479, p = 0.018) or OT-E process skills (R = −0.516, p = 0.001) scores as well as between lFEF and TMT/B-A scores (R = −0.423, p = 0.040) were also found in the study group. Furthermore, significant positive correlations were found between strength of functional connectivity in PCC and global prose memory (R = 0.498, p = 0.013), immediate recall (R = 0.407, p = 0.048), delayed recall (R = 0.408, p = 0.048) scores, and between lFEF and immediate recall scores (R = 0.478, p = 0.018). In contrast, we did not find significant correlations between neuropsychological/occupational and fMRI data in the control group, thereby lending support to the idea that all the observed changes in the treated group were driven by CT exposure and not by the six month time interval. A detailed description of correlation analysis is shown in Supporting Information, Table S4.

### Cortical Thickness

**Figure 4:**
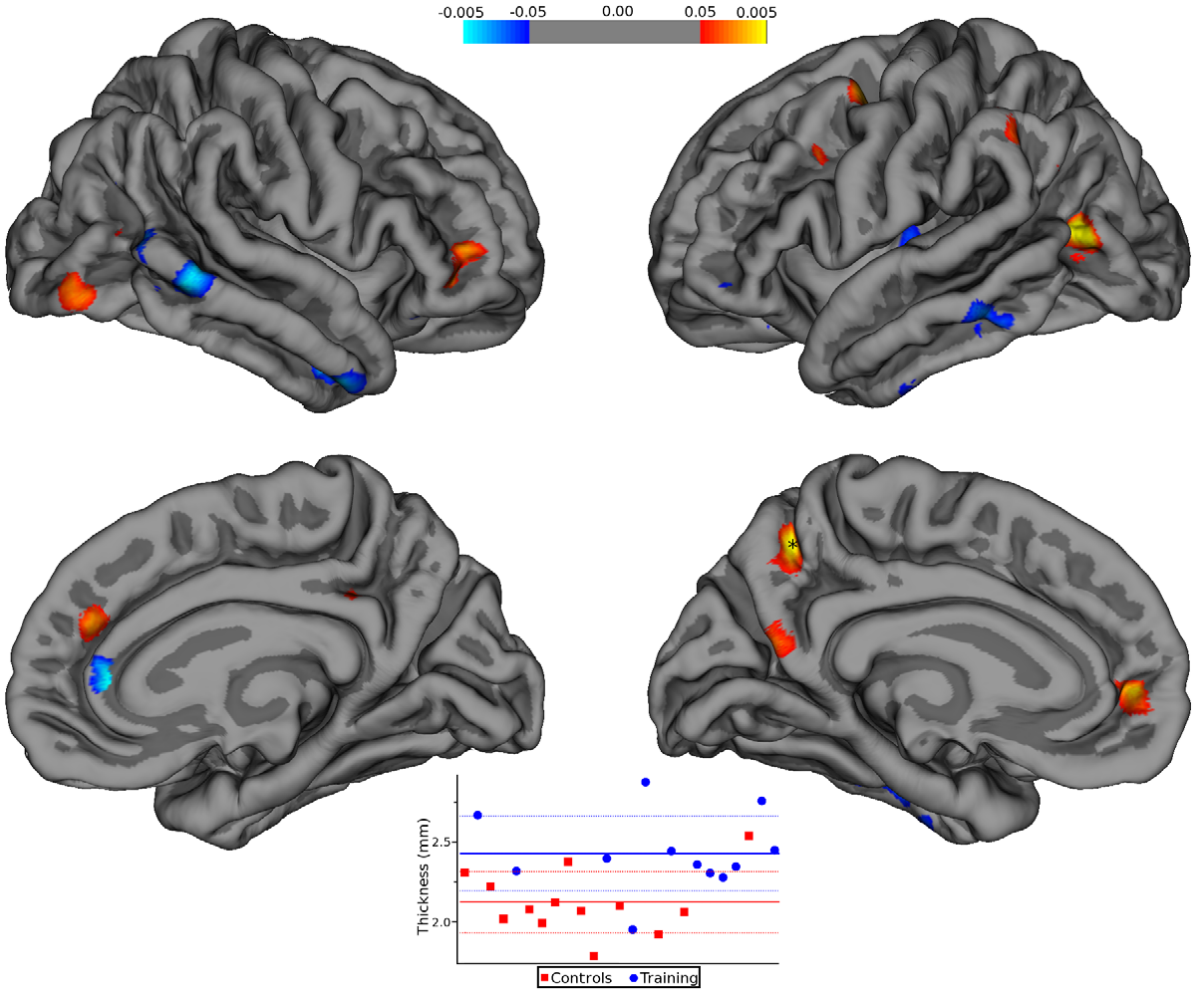
CT positively affects cortical thickness. Statistical p-maps, employing a threshold ranging from ±0.05 to ±0.005 (uncorrected), depict brain areas that showed a significant change in cortical thickness. In the right hemisphere, these areas are located in the middle temporal, rostral anterior cingulated, pars orbitalis, superior frontal, supramarginal, lateral occipital, isthmus cingulate, superior temporal, and lateral orbitofrontal. In the left hemisphere, these areas are located in the inferior parietal, precuneus, inferior temporal, superior frontal, caudal middle frontal, middle temporal, supramarginal, insula, lateral occipital, pars orbitalis, and inferior parietal. The plot is the graphical distribution of the average thickness for all subjects at the point indicated with a star in the lower left panel. Each red square represents a subject in the control group and the blue square a subject in the training group. The heavy dashed lines are the group means and the lighter dashed lines standard deviations. The pseudocolor bar graphically shows the extension of differences in cortical thickness scores.

Two subjects from the training group were excluded from analysis due to withdraw prior to the second scanning session. Two time-point longitudinal analysis modelling vertex-wise difference maps with group as two separate planes (two levels of a single discrete factor) with one continuous factors as covariate (age) yielded several areas with statistically significant differences in cortical thickness between the two groups (Fig. 4). No effects were seen for genotype expression. We did not find significant correlations between cortical thickness and fMRI data in both groups. A detailed description of correlation analysis results is shown in Supporting Information, Table S5.

### Dopamine-related Gene Polymorphisms

Five different dopamine receptors subtypes, *DRD1–5*, have been cloned and characterized so far. Dopaminergic neurotransmission is also regulated by the catechol-O-methyltransferase gene (*COMT*; coding for the enzyme that catabolizes dopamine) and by the dopamine transporter gene (*DAT1*; coding for the protein responsible for dopamine reuptake). As dopaminergic neurotransmission affects cognition and behavior, in this part of the study we tested the hypothesis that specific dopamine-related gene polymorphisms may differently modulate the individual response to CT.

**Figure 5:**
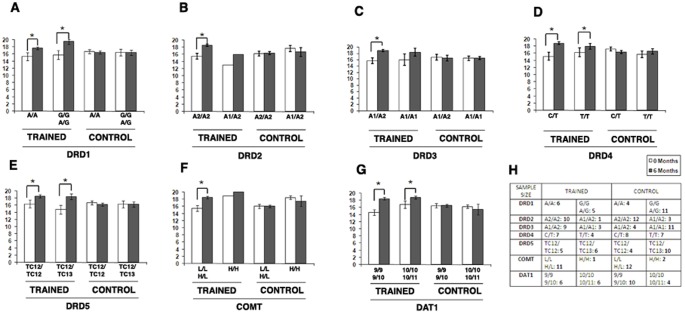
Influence of dopaminergic gene functional polymorphisms on OT-E process skills performance. Bar graphs show the results of the OT-E Process Skills performance in study participants expressing different dopamine-related gene polymorphisms. No differences were found among carriers of different *DRD1* (**A**), *DRD2* (**B**), *DRD4* (**D**)**,** *DRD5* (**E**)**,** and *DAT1* (**G**) polymorphisms in the trained group. Compared to individuals carrying the *DRD3 A1/A1(Ser/Ser)* genotype, *DRD3 A1/A2(Ser/Gly)* carriers showed an increased response to CT (**C**). *COMT L/L-H/L (Val/Val-Val/Met)* carriers also benefitted the most from CT (**F**). Table (**H**) shows sample size.

To that aim, we investigated DRD1, DRD2, DRD3, DRD4, DRD5, COMT and DAT1. Each participant was screened for the following polymorphisms: DRD1–48 A/G, DRD2 TAQ-IA, DRD3 Ser9Gly, DRD4 521C/T, DRD5 (TC)n, COMT Val158Met and DAT1 40 VNTR in the 3‘ untranslated region (Fig. 5).

When, both in the control and trained group, we matched the results of neuropsychological/occupational tests with the investigated dopamine-related polymorphisms, no differences were found with the exception of performance in OT-E process skills in trained individuals carrying specific *DRD3* and *COMT* genotypes.

When evaluating *DRD3 ser9gly (Ser = A1* allele*; Gly = A2* allele*)* and *COMT Val158Met* (*Met = H* allele*; Val = L* allele) variants, we found that, after CT, individuals jointly carrying the *DRD3 Ser/Gly* and *COMT Val/Val-Val/Met* genotypes performed better in OT-E process skills (mean score for DRD3∶18.7±0.36 SEM; p<0.006 Paired t-test; COMT: 18.5±0.38 SEM; p<0.001 Paired t-test) when compared to carriers of the *Ser/Ser* and *Met/Met* genotypes (Fig. 5C, 5F). No differences were found in the control group.

## Discussion

The enriched environment is a combination of complex inanimate and social stimuli [39]. This definition implies that the relevance of a single contributing factor cannot be isolated but, on the contrary, interaction of all variables is essential to produce an enriching environment. As shown in animal models, aerobic exercise and enriched environment can enhance learning capability, promote neurogenesis, and favor synaptic plasticity [40], [41]. Human studies also indicate that aerobic exercise promotes structural and functional brain changes that are critical to maintain higher-level cognitive functions. Even though aerobic exercise has, by itself, strong beneficial effects on brain trophism and cognition [41], addition of cognitive stimuli can further enhance these beneficial effects [42]. In animal models, hippocampal neurogenesis, one of the mechanisms induced by aerobic exercise, is enhanced and reinforced by cognitive activities [43]. It should be emphasized that strong evidence indicates [44] that the brain has a neural and cognitive reserve that can set in motion a range of functional compensations that counteract the cognitive decline associated with diseases (dementia, trauma) or aging, thereby providing a potential for lifelong cognitive flexibility and adaptability [41].

We chose a training program designed to simultaneously stimulate different cognitive, motor, and sensorial domains in order to maximize effects on cognitive reserve.

The main findings of the study indicate that CT is beneficial on cognition (memory skills) and on the modulation of functional connectivity (DMN and DAN) and that these results translate in an overall better performance in ADL activities [45]–[47]. Our results are in line with recent reports indicating that multimodal enrichment can promote brain plasticity, thereby counteracting age-dependent cognitive and behavioral decline as well as brain atrophy [1], [5], [6].

Our fMRI results indicate a rearrangement of RSNs that, overall, results in concomitant increase or decrease strength of functional connectivity within different network regions. These modifications can be interpreted as CT-driven mechanisms set in place to modify cognitive and occupational abilities [48]–[50]. BOLD data analysis within the study group showed modification of DMN and DAN connectivity suggesting that CT can promote functional network reorganization in a relatively short time period. This reorganization is associated with an overall significant increase in cognitive performances as confirmed by correlation analysis (Supporting Information, Table S4). These findings lead us to speculate that functional reorganization can promote beneficial cognitive/occupational effects.

DMN activity is associated with internal mental processes. Several fMRI studies have shown that aging induces a reduction in DMN connectivity [51]. Recent studies also indicated that DMN areas (especially PCC and PrC) are not stable in elderly subjects, a potentially relevant patho-physiological phenomenon as these specific regions are involved in episodic memory, a function that declines with aging [52]. Furthermore, impaired connectivity within posterior midline DMN regions correlates with the development of memory deficits, a phenomenon also called “retrosplenial amnesia” [53] that it is present in subjects showing early signs of mild cognitive impairment (MCI) or AD. These posterior DMN regions are also particularly vulnerable to early deposits of beta-amyloid (Aβ) [54] which is thought to promote decreased connectivity. Aβ deposition is crucial to trigger memory deficits in AD patients. The process is relevant to brain aging as 10% of non-demented elderly individuals has this pattern of amyloid accumulation [55], [56] shows impaired DMN connectivity and decline of working memory [57].

Our fMRI results indicate a CT-driven functional reorganization of DMN connectivity at T6, which involves decreased PrC activity and increased PCC activity in the trained group [49], [50]. These changes are potentially important as aging is associated with an overall decrease of DMN connectivity especially within the PCC [17], a modification that is considered prodromal of AD [18]. Extended exposure to multimodal activities that promotes, in a AD-targeted region, enhanced cognition and ADL capabilities as well as maintaining of functional connectivity is a potential valuable therapeutic tool to delay AD progression.

Our fMRI findings are in agreement with the neuropsychological/occupational results which show a better performance in memory tests and OT-E in the trained group, thereby further substantiating the idea of a functional relationship between connectivity changes and increased cognitive functioning. In fact, correlation analysis between BOLD data and neuropsychological/occupational test scores show a significant correlation in which decreased strength of functional connectivity in PrC is matched with better performances in delayed recall and process skills. This correlation suggests that CT rearranges functional connectivity within the DMN and promotes higher levels of cognitive performance. The fact that a positive neuropsychological effect was also linked to lower PrC activity is not completely surprising as increased activation of a specific brain area is not always beneficial [see for instance the unbalanced hyper-activation found in different hippocampal areas in patients affected by schizophrenia, depression or post-traumatic stress disorder (PTSD) [58] ]. We also found significant positive correlations between PCC activity and scores of global prose memory and its subtests. These correlations may be relevant for their patho-physiological implications [8].

DAN connectivity has been associated with voluntary re-orienting of attention and is typically triggered by cognitive tasks [59]. A better attentive performance should therefore result in higher connectivity of the network and we, indeed, detected a better integration within DAN areas in the treated group. CT affects functioning of the lFEF area which is crucial in controlling attention [59]. In fact, we found significant correlations between the strength of functional connectivity in lFEF and TMT/B-A scores. In other words, increased activation of this area positively correlates with performance expressed here in terms of time to complete the task.

Our exploratory results show that CT can affect a positive rearrangement of functional connectivity and higher cognitive functions, but as described in the literature [42], [60], the correlation between single components of the training and their effects on specific networks is difficult to identify. We favor the hypothesis that effects we report on cognition are determined by the combination of all variables [43]. These findings warrant future studies investigating a larger cohort of aging individuals.

Analysis of cortical thickness changes between the two groups showed modification in regions like the orbitofrontal cortex (OFC), PrC and selected regions of the temporal lobe especially in the Hp which are located within DMN and DAN. With correlation analysis we tested the hypothesis that changes in functional connectivity can be linked to underlying modifications of cortical thickness. However, no significant correlations were found, thereby suggesting that CT promotes functional and structural changes that are not directly related.

Recent studies indicate that mental and aerobic exercises can counteract age-related cognitive decline and reduce the risk of dementia [1] by promoting functional and neurochemical brain changes that lead to plastic structural reorganization [61]. Our results are in agreement with this notion as they indicate structural changes in brain areas that are involved in short-term memory consolidation [62], a process that is mediated by dopamine receptors. Cortical thickness results also indicate increased thickness in posterior regions. Buckner and colleagues [8] have shown that the Hp, a key region for long-term memory (found ameliorated in the trained group), also mediates the physiological functioning of the two areas, PCC and PrC, that we found increased in their thickness upon CT exposure. We therefore hypothesize that CT may enhance cognitive performances and promote functional reorganization of neural networks that ultimately and indirectly favors modification in cortical thickness in areas that are related to memory circuits. Overall these results suggest a cerebral structural reorganization that can be useful in counteracting age- or AD- dependent thinning of the cortex. These intriguing results require confirmation in a larger study population.

Evaluation of the influence of dopamine-related genes on the response to CT, with the limitations related to the small size of our sample, show that the joint presence of *DRD3* (*Ser9Gly*) and *COMT* (*Val158Met*) polymorphisms is likely associated with changes in functional connectivity and the capability to maximize positive effects of the training. These results are in line with the fact that the dopaminergic system is a key modulator of cognition and its impairment plays an important role in the development of age-related cognitive decline [19]. Moreover, it is important to note that the two genes appear to have a synergistic action as all the individuals who gained the most from CT were simultaneously carriers of the *DRD3 Ser/Gly* and the *COMT Val/Val*, *Val/Met* genotypes.

*DRD3* is an inhibitory receptor and considered to be involved in the modulation of dopamine-dependent prefrontal functions [63]. One of the most investigated polymorphism is the *Ser9Gly* that is associated with lower receptor affinity for dopamine, thereby producing a decreased inhibitory dopaminergic tone. Contrary to what we found in response to CT, where the presence of *DRD3 Ser9Gly* seems to be advantageous, a large number of studies (but not all) have linked the presence of the *Gly* allele with a high risk for schizophrenia, ADHD, substance abuse as well as the development of attention and working memory deficits [64]. However, an association between the *Gly* allele and improved social interactions has also been suggested [65].

*COMT* is the main mammalian enzyme involved in the extracellular degradation of synaptically released dopamine. The *COMT* gene has a functional variant in which substitution of valine (*Val*) for methionine (*Met*) at codon *158* (*Val158Met*) occurs. The *Met* allele has been associated with low enzymatic activity, thereby producing higher levels of dopamine in the prefrontal cortex. Conversely, the *Val* allele is associated with higher enzymatic activity and lower levels of prefrontal dopamine [66]. In humans, dopamine transmission is closely linked with enhanced cognitive performance (working memory, attention, fluid intelligence, etc), a phenomenon exerted by positive modulation of the activity of the prefrontal cortex. In a recent study, *COMT* polymorphism *Val/Val,* analyzed in a cohort of healthy subjects, was found to be more sensitive to stress-related epigenetic changes and associated with more efficient working memory performance [67], [68].

Translation of these findings to our results leads to an intriguing hypothesis: the increased cognitive functioning found in individuals carrying the *COMT Val/Val* and *Val/Met* polymorphisms may be associated with a higher sensitivity to changes that reduce the enzymatic activity, thereby favoring increased dopamine levels in the PCC. Supporting this hypothesis, a recent study [69] suggested that the *COMT Val/Val* genotype favourably modulates PCC activity, thereby providing a rationale for our DMN results.

In conclusion, our findings lend support to the idea that multimodal enrichment can promote morpho-functional changes, enhances cognitive and occupational performances in the elderly. We also show that specific *DRD3* and *COMT* polymorphisms may increase the positive CT effects. A major limitation of this study is the relatively small number of investigated individuals making the genotyping results largely exploratory at this point. These findings need to be validated in a larger study cohort.

Surprisingly, CT-driven beneficial effects can be seen on a short time scale of six months.

The molecular determinants underlying the process are likely to be multiple, but a major role can be speculated for the Brain Derived Neurotrophic Factor (BDNF). In that respect, aerobic exercise has been shown to increase BDNF levels and promote changes in the hippocampus size in elderly individuals [1]. Furthermore, recent evidence, in animal models [70], indicates that altered BDNF/TrkB (tyrosine kinase B) signalling, triggered by behavioral manipulations, can favor molecular modifications (i.e.: synaptic tagging) that are critical to start memory consolidation.

Further studies will identify the specific roadmap of all the mechanisms set in motion by multimodal stimulations; however, our findings suggest that engagement in these activities can be a rapidly effective and largely inexpensive strategy to promote neural and cognitive modifications.

## Supporting Information

Table S1 **fMRI statistical analysis results.**
(DOC)

Table S2 **Psychometric and Occupational Therapy test results.** Psychometric and Occupational Therapy test* results. Data are shown as mean and median for the trained and control groups before (t0) and after (t6) completion of the training.
(DOC)

Table S3 **Results of two factor ANOVA followed by Tukey HSD post-hoc test for neuropsychological evaluations.** Results of two factor ANOVA followed by Tukey HSD post-hoc test for neuropsychological evaluations. Data are p values. Significant p values (95% confidence level) are indicated in bold. Data showed underlined are p values significant at p<0.1 (90% confidence level).
(DOC)

Table S4 **Correlation analysis between neuropsychological and fMRI variables.** Correlation analysis between neuropsychological and fMRI variables. Data are correlation coefficients (R) and p values from Spearman correlation analysis. p values lower than 0.05 and the corresponding correlation coefficients are indicated in bold.
(DOC)

Table S5 **Correlation analysis between fMRI data and cortical thickness scores.** Correlation analysis between fMRI data and cortical thickness scores. Data are shown as correlation coefficients (R) and p values from Spearman correlation analysis.
(DOC)

Text S1 **Combination Training: tasks.**
(DOC)
