# Supplementary material for: Combination Training in Aging Individuals Modifies Functional Connectivity and Cognition, and Is Potentially Affected by Dopamine-Related Genes
Source: PLoS One. 2012 Aug 28;7(8):e43901. doi: 10.1371/journal.pone.0043901 (PMC3429431; doi:10.1371/journal.pone.0043901)
Supplement: Table S1 — fMRI statistical analysis results. (DOC) [file pone.0043901.s001.doc]

**Table S1**

**fMRI statistical analysis results**

|  |  |  | |  | |  | | TTEST ‐ T0/T6 | | TTEST ‐ T0/T6 | |
| --- | --- | --- | --- | --- | --- | --- | --- | --- | --- | --- | --- |
|  |  | ANOVA ‐ group  factor | | ANOVA ‐ time  factor | | ANOVA ‐ interaction | | trained  group | | control  group | |
|  | Coordinates | FSCORE | P | FSCORE | P | FSCORE | P | TSCORE | P | TSCORE | P |
| **RSN1** |  |  |  |  |  |  |  |  |  |  |  |
| Precuneus | (1, ‐52, 22) | 12,4183 | 0,0009 | 1,5715 | 0,2161 | 4,5359 | 0,0383 | 4,7988 | 0,0001 | 0,8627 | 0,3969 |
| Right Angular Gyrus | (53, ‐52, 19) | 2,2158 | 0,1431 | 1,2459 | 0,2699 | 4,3141 | 0,0432 | 2,3562 | 0,027 | ‐0,45 | 0,6567 |
| Posterior Cingulate Cortex | (‐1, ‐20, 30) | 1,7922 | 0,187 | 1,523 | 0,2232 | 4,5293 | 0,0385 | ‐2,7212 | 0,0119 | 0,5121 | 0,6133 |
| **RSN2** |  |  |  |  |  |  |  |  |  |  |  |
| Left Frontal Eye Field | (‐33, 15, 39) | 1,7194 | 0,196 | 0,0966 | 0,7573 | 4,4547 | 0,04 | ‐2,2737 | 0,0322 | 0,6069 | 0,5496 |
