# Supplementary material for: Combination Training in Aging Individuals Modifies Functional Connectivity and Cognition, and Is Potentially Affected by Dopamine-Related Genes
Source: PLoS One. 2012 Aug 28;7(8):e43901. doi: 10.1371/journal.pone.0043901 (PMC3429431; doi:10.1371/journal.pone.0043901)
Supplement: Table S2 — Psychometric and Occupational Therapy test results. Psychometric and Occupational Therapy test* results. Data are shown as mean and median for the trained and control groups before (t0) and after (t6) completion of the training. (DOC) [file pone.0043901.s002.doc]

Table S2

|  |  | **Training** | | | | | **Control** | | | | |
| --- | --- | --- | --- | --- | --- | --- | --- | --- | --- | --- | --- |
|  |  |  |  |  |  | Interquartile |  |  |  |  | Interquartile |
| **Test** | **Time** | Mean | SD^a^ | SEM^b^ | Median | Range^c^ | Mean | SD^a^ | SEM^b^ | Median | Range^c^ |
| Mini Mental State Examination |  |  |  |  |  |  |  |  |  |  |  |
| (MMSE) | t0 | 29 | 2 | 0.5 | 29 | 3 | 29 | 2 | 0.44 | 30 | 2 |
|  | t6 | 29 | 2 | 0.4 | 29 | 1 | 28 | 2 | 0.52 | 29 | 3 |
| Trial Making Test |  |  |  |  |  |  |  |  |  |  |  |
| (TMT/A) | t0 | 65 | 20 | 5.2 | 65 | 32 | 58 | 41 | 10.54 | 43 | 35 |
|  | t6 | 59 | 26 | 6.9 | 59 | 31 | 54 | 27 | 6.94 | 46 | 33 |
| Trial Making Test |  |  |  |  |  |  |  |  |  |  |  |
| (TMT/B) | t0 | 111 | 48 | 12.8 | 111 | 71 | 79 | 37 | 9.54 | 79 | 66 |
|  | t6 | 97 | 43 | 11.5 | 97 | 57 | 77 | 30 | 7.68 | 79 | 24 |
| Trial Making Test |  |  |  |  |  |  |  |  |  |  |  |
| (TMT/B-A) | t0 | 46 | 34 | 9.1 | 46 | 39 | 22 | 58 | 15.05 | 32 | 57 |
|  | t6 | 38 | 29 | 7.6 | 38 | 30 | 28 | 12 | 3.03 | 29 | 19 |
| Babcock Story Recall Test | t0 | 10 | 3 | 0.8 | 10 | 4 | 11 | 3 | 0.86 | 10 | 5 |
|  | t6 | 13 | 3 | 0.7 | 13 | 4 | 13 | 3 | 0.65 | 13 | 5 |
| Immediate recall |  |  |  |  |  |  |  |  |  |  |  |
| (Bi) | t0 | 6 | 1 | 0.4 | 6 | 2 | 6 | 1 | 0.37 | 6 | 2 |
|  | t6 | 7 | 1 | 0.4 | 7 | 2 | 7 | 1 | 0.36 | 7 | 3 |
| Delayed recall |  |  |  |  |  |  |  |  |  |  |  |
| (Bd) | t0 | 6 | 2 | 0.4 | 6 | 3 | 6 | 1 | 0.38 | 6 | 3 |
|  | t6 | 7 | 1 | 0.3 | 7 | 2 | 7 | 1 | 0.33 | 7 | 2 |
| Frontal Assessment Battery |  |  |  |  |  |  |  |  |  |  |  |
| (FAB) | t0 | 17 | 2 | 0.4 | 17 | 3 | 17 | 1 | 0.33 | 18 | 2 |
|  | t6 | 17 | 1 | 0.3 | 17 | 2 | 17 | 2 | 0.44 | 17 | 3 |
| Phonemic fluency test |  |  |  |  |  |  |  |  |  |  |  |
| (FAS) | t0 | 31 | 10 | 2.6 | 31 | 8 | 33 | 9 | 2.42 | 34 | 16 |
|  | t6 | 32 | 10 | 2.8 | 32 | 16 | 34 | 9 | 2.27 | 36 | 8 |
| Motor Skills | t0 | 15 | 1 | 0.3 | 15 | 1 | 15 | 2 | 0.45 | 16 | 1 |
|  | t6 | 15.8 | 0.8 | 0.2 | 16 | 0 | 15 | 2 | 0.50 | 15 | 1 |
| Process | t0 | 16 | 2 | 0.7 | 16 | 4 | 17 | 2 | 0.56 | 17 | 3 |
|  | t6 | 19 | 1 | 0.3 | 19 | 2 | 16 | 2 | 0.40 | 16 | 3 |
| Time | t0 | 1.7 | 0.8 | 0.2 | 1.7 | 1 | 1.9 | 0.6 | 0.16 | 2 | 1 |
|  | t6 | 1.2 | 0.5 | 0.1 | 1.2 | 0.3 | 1.7 | 0.6 | 0.15 | 1.5 | 1 |

^a^ SD=standard deviation

^b^ SEM=standard error of the mean

^c^ Calculated as the difference between the upper and lower quartiles

***Occupational Therapy test**

OT-E is a simplified version of the Assessment Motor and Process Skills (AMPS) which evaluates performance in activities of daily living (ADL) including of motor and process skills. OT-E can be administered by occupational therapists allowing an assessment of the same domains of AMPS, in fact it offers a way of thinking about and evaluating performance in ADL within the same conceptual framework of AMPS (Fisher, 2006, 2009). The basic assumptions is: the performance of the subject, which assesses the OT during the course of the ADL, is composed of motor (OT-E Motor Skills) and process skills (OT-E-Process Skills), occurring with different reaction times (OT-E Time). The OT-E evaluates how the performance of the subject is manifested in ADL taking into account the individual reaction time and how motor and process skills improve/limit performance. The OT-E is divided into subtests like the OT-E Motor Skills, the OT-E Process Skills, as well as, the OT-E Time. The OT-E is used to measure the performance in ADL that is assessed by rating the effort, efficiency, safety, and independence in 16 motor skills and 20 process skill items, while the person is performing ADL tasks. The OT-E Motor Skills is the subtest that measures the ability to move and interact with the task, objects and environment. In this subtest there are different domains such as: Posture (stabilizes, align, position), mobility (walks, reaches, bends), coordination (coordination, manipulation, fluidity), strength and stress (moving, lifting, carrying, calibrates, grabs), energy (resistance, cadence). The OT-E Process Skills is the subtest assessing the ability used in managing and modifying the actions during ADL (Fisher and Kielhofner, 1995). In this subtest there are different domains such as: Energy (cadence, assists), Knowledge (choose, use, handling, be careful, informed), temporal organization (begins, continues, run in succession, ends), organization of space and objects (search /find, collect, organize, restore, navigate), adaptation (note/answers, right, system, benefits). The OT-E Time is the readiness/reaction times of the subject during ADL.
