# Supplementary material for: Combination Training in Aging Individuals Modifies Functional Connectivity and Cognition, and Is Potentially Affected by Dopamine-Related Genes
Source: PLoS One. 2012 Aug 28;7(8):e43901. doi: 10.1371/journal.pone.0043901 (PMC3429431; doi:10.1371/journal.pone.0043901)
Supplement: Table S3 — Results of two factor ANOVA followed by Tukey HSD post-hoc test for neuropsychological evaluations. Results of two factor ANOVA followed by Tukey HSD post-hoc test for neuropsychological evaluations. Data are p values. Significant p values (95% confidence level) are indicated in bold. Data showed underlined are p values significant at p<0.1 (90% confidence level). (DOC) [file pone.0043901.s003.doc]

**Table S3**

|  | Two factor ANOVA, p | | | Tukey HSD post-hoc test, p | | | | | |
| --- | --- | --- | --- | --- | --- | --- | --- | --- | --- |
| Test | Time | Group | Time*Group | Control (baseline)  *vs*  Trained (baseline) | Control (baseline)  *vs*  Control (six months) | Control (baseline)  *vs*  Trained (six months) | Trained (baseline)  *vs*  Control (six months) | Trained (baseline)  *vs*  Trained (six months) | Control (six months)  *vs*  Trained (six months) |
| Mini Mental State Examination (MMSE) | 0.604 | 0.614 | 0.370 | 0.781 | 0.310 | 0.992 | 0.470 | 0.792 | 0.323 |
| Trial Making Test (TMT/A) | 0.519 | 0.456 | 0.887 | 0.531 | 0.717 | 0.943 | 0.327 | 0.585 | 0.669 |
| Trial Making Test (TMT/B) | 0.415 | **0.016** | 0.569 | 0.149 | 0.998 | 0.648 | 0.104 | 0.768 | 0.538 |
| Trial Making Test (TMT/B-A) | 0.902 | 0.083 | 0.465 | 0.299 | 0.971 | 0.653 | 0.544 | 0.932 | 0.885 |
| Babcock Story Recall Test | **0.005** | 0.766 | 0.383 | 0.839 | 0.467 | 0.263 | 0.118 | 0.051 | 0.976 |
| Immediate recall (BI) | **0.015** | 0.271 | 0.317 | 0.445 | 0.702 | 0.755 | 0.062 | 0.080 | 1.000 |
| Delayed recall (BD) | **0.006** | 0.716 | 0.184 | 0.623 | 0.687 | 0.294 | 0.111 | **0.024** | 0.900 |
| Frontal Assessment Battery (FAB) | 0.792 | 0.522 | 0.490 | 0.348 | 0.493 | 0.523 | 0.790 | 0.766 | 0.971 |
| Phonemic fluency test (FAS) | 0.658 | 0.435 | 0.985 | 0.590 | 0.740 | 0.810 | 0.388 | 0.768 | 0.571 |
| OT-E Motor Skills | 0.575 | 0.109 | 0.368 | 0.611 | 0.806 | 0.127 | 0.454 | 0.311 | 0.078 |
| OT-E Process | **0.006** | 0.165 | **0.004** | 0.666 | 0.999 | **0.019** | 0.722 | **0.001** | **0.014** |
| OT-E Time | 0.070 | **0.045** | 0.335 | 0.450 | 0.531 | **0.008** | 0.888 | 0.055 | **0.037** |
