# Supplementary material for: Combination Training in Aging Individuals Modifies Functional Connectivity and Cognition, and Is Potentially Affected by Dopamine-Related Genes
Source: PLoS One. 2012 Aug 28;7(8):e43901. doi: 10.1371/journal.pone.0043901 (PMC3429431; doi:10.1371/journal.pone.0043901)
Supplement: Table S4 — Correlation analysis between neuropsychological and fMRI variables. Correlation analysis between neuropsychological and fMRI variables. Data are correlation coefficients (R) and p values from Spearman correlation analysis. p values lower than 0.05 and the corresponding correlation coefficients are indicated in bold. (DOC) [file pone.0043901.s004.doc]

**Table S4**

|  | **Trained Group** | | **Control Group** | |
| --- | --- | --- | --- | --- |
| **Pair of variables** | **Spearman R** | **p** | **Spearman R** | **p** |
| PrC & MMSE | 0.110 | 0.608 | 0.238 | 0.263 |
| PrC &TMT/A | 0.089 | 0.680 | 0.007 | 0.975 |
| PrC &TMT/B | 0.211 | 0.322 | -0.297 | 0.159 |
| PrC &TMT/B-A | 0.183 | 0.393 | 0.006 | 0.977 |
| PrC & Global Prose Memory | -0.313 | 0.136 | -0.171 | 0.424 |
| PrC & Immediate Recall | -0.277 | 0.189 | -0.126 | 0.556 |
| PrC & Delayed Recall | **-0.430** | **0.036** | -0.163 | 0.448 |
| PrC & FAS | -0.176 | 0.411 | -0.123 | 0.568 |
| PrC & FAB | -0.273 | 0.196 | -0.291 | 0.167 |
| PrC & OT-E Motor skills | -0.199 | 0.350 | -0.355 | 0.088 |
| PrC & OT-E Process skills | **-0.547** | **0.006** | -0.176 | 0.412 |
| PrC & OT-E Time | 0.275 | 0.193 | 0.393 | 0.057 |
| rAg & MMSE | 0.052 | 0.810 | 0.173 | 0.420 |
| rAg &TMT/A | -0.037 | 0.862 | 0.109 | 0.613 |
| rAg &TMT/B | -0.039 | 0.857 | -0.095 | 0.658 |
| rAg &TMT/B-A | -0.054 | 0.802 | 0.154 | 0.472 |
| rAg & Global Prose Memory | -0.381 | 0.067 | -0.282 | 0.182 |
| rAg & Immediate Recall | -0.404 | 0.050 | -0.199 | 0.352 |
| rAg & Delayed Recall | **-0.479** | **0.018** | -0.344 | 0.099 |
| rAg & FAS | -0.104 | 0.629 | -0.226 | 0.288 |
| rAg & FAB | -0.202 | 0.345 | -0.281 | 0.184 |
| rAg & OT-E Motor skills | -0.293 | 0.164 | -0.275 | 0.194 |
| rAg & OT-E Process skills | **-0.517** | **0.010** | -0.276 | 0.191 |
| rAg & OT-E Time | 0.162 | 0.449 | 0.379 | 0.068 |
| PCC & MMSE | -0.192 | 0.368 | 0.168 | 0.433 |
| PCC &TMT/A | -0.340 | 0.104 | 0.211 | 0.323 |
| PCC &TMT/B | -0.328 | 0.118 | 0.291 | 0.167 |
| PCC &TMT/B-A | -0.267 | 0.207 | 0.161 | 0.451 |
| PCC & Global Prose Memory | **0.498** | **0.013** | -0.038 | 0.861 |
| PCC & Immediate Recall | **0.407** | **0.049** | -0.027 | 0.899 |
| PCC & Delayed Recall | **0.408** | **0.048** | -0.017 | 0.936 |
| PCC & FAS | 0.075 | 0.727 | **-0.431** | **0.035** |
| PCC & FAB | 0.007 | 0.976 | -0.052 | 0.809 |
| PCC & OT-E Motor skills | 0.109 | 0.612 | -0.038 | 0.861 |
| PCC & OT-E Process skills | 0.378 | 0.069 | -0.275 | 0.193 |
| PCC & OT-E Time | 0.184 | 0.390 | 0.305 | 0.148 |
| LFEF & MMSE | 0.074 | 0.730 | 0.358 | 0.086 |
| LFEF &TMT/A | -0.215 | 0.313 | 0.074 | 0.732 |
| LFEF &TMT/B | -0.354 | 0.090 | -0.181 | 0.397 |
| LFEF &TMT/B-A | **-0.423** | **0.040** | 0.011 | 0.958 |
| LFEF & Global Prose Memory | **0.478** | **0.018** | 0.009 | 0.966 |
| LFEF & Immediate Recall | 0.384 | 0.064 | 0.020 | 0.926 |
| LFEF & Delayed Recall | 0.306 | 0.146 | -0.055 | 0.800 |
| LFEF & FAS | -0.051 | 0.812 | 0.039 | 0.857 |
| LFEF & FAB | -0.132 | 0.538 | -0.039 | 0.855 |
| LFEF & OT-E Motor skills | 0.198 | 0.353 | -0.355 | 0.088 |
| LFEF & OT-E Process skills | 0.297 | 0.159 | -0.189 | 0.376 |
| LFEF & OT-E Time | 0.034 | 0.876 | 0.150 | 0.485 |
