# Supplementary material for: Combination Training in Aging Individuals Modifies Functional Connectivity and Cognition, and Is Potentially Affected by Dopamine-Related Genes
Source: PLoS One. 2012 Aug 28;7(8):e43901. doi: 10.1371/journal.pone.0043901 (PMC3429431; doi:10.1371/journal.pone.0043901)
Supplement: Table S5 — Correlation analysis between fMRI data and cortical thickness scores. Correlation analysis between fMRI data and cortical thickness scores. Data are shown as correlation coefficients (R) and p values from Spearman correlation analysis. (DOC) [file pone.0043901.s005.doc]

**Table S5**

**Correlation analysis between fMRI data and cortical thickness scores.**

|  | **Trained Group** | | **Control Group** | |
| --- | --- | --- | --- | --- |
| **Pair of variables** | **Spearman R** | **p** | **Spearman R** | **p** |
| PrC_BOLD & PrC_Thickness | 0.503 | 0.138 | -0.045 | 0.894 |
| rAg_BOLD & rAg_Thickness | 0.358 | 0.310 | 0.136 | 0.689 |
| PCC_BOLD & PCC_Thickness | -0.103 | 0.777 | -0.091 | 0.790 |
| LFEF_BOLD & lFEF_Thickness | 0.176 | 0.626 | 0.355 | 0.285 |
