# Supplementary material for: Combination Training in Aging Individuals Modifies Functional Connectivity and Cognition, and Is Potentially Affected by Dopamine-Related Genes
Source: PLoS One. 2012 Aug 28;7(8):e43901. doi: 10.1371/journal.pone.0043901 (PMC3429431; doi:10.1371/journal.pone.0043901)
Supplement: Text S1 — Combination Training: tasks. (DOC) [file pone.0043901.s006.doc]

Text S1

**Combination training: tasks**

The combination training we employed was structured as a detailed program of activities organized to favor multimodal stimulation. To that aim, it included three different areas: 1) brain training, a set of activities to stimulate higher cognitive functions; 2) aerobic training, and 3) exposure to musical stimulation (see Table 1).

Training lasted for six months: activities of the last three months were designed to be more difficult and engaging.

Combination Training was performed at home. In addition, study group participants attended a “fun-recreation project” in which they were set to monitor the training, discuss, and organize strategies to overcome difficulties which emerged from the execution of specific activities. This meeting was organized so as to provide the subjects with a period of group multimodal stimulation.

Each volunteer was provided with a kit containing: a brochure describing cognitively stimulating activities, pens, “Supermind” musical compilation (a selected list of musical pieces that included music from Vivaldi, Chopin, Debussy, Mozart, Wagner, Queen, Elvis Presley, and The Rolling Stones) and dance music. Book topics included history, culture, religion and fiction. Once volunteers finished a book, they exchanged them.

The brochure contained a detailed description of each activity (crossword puzzles, sudoku and word puzzles for the first three months; logical puzzle grids, sudoku and games named “transfer“ for the last three months). For each given activity the subject was asked to write the date of execution, the time dedicated to the activity (1 hour minimum), and to highlight all difficulties encountered.

Subjects were asked to turn on the radio and insert the CD “Supermind” compilation every time they decided to engage in Brain Training activities.

The weekly schedule described in the brochure was as follow:

- Monday: 1 hour of crossword while listening to musical stimulation (CD compilation of supermind) + 1 hour of activity of daily living (ADL): walking;
- Tuesday: 1 hour of reading a book (at least 30 pages) immediately followed by writing a brief summary of what was read;
- Wednesday: 1 hour of Sudoku while listening to musical stimulation (CD compilation of supermind)] + 1 hour of ADL;
- Thursday: 1 hour to prepare a structured discussion or to prepare common activities for the group;
- Friday: 1 hour of word puzzles while listening to musical stimulation (CD compilation of supermind)] + 1 hour ADL: (dance using dance CD);
- Saturday: 2 hours of "fun-recreation project" alternated with rest;
- Sunday: no planned activities.

Overall, the tasks were chosen to be easy to perform and allowed us to provide instructions entire study group simultaneously in a joint session.

The fun-recreation project took place every fourteen days, during this project the groups met and expressed the strengths and weaknesses related to the course of program, they also elaborated strategies to simplify the more difficult brain training sessions (i.e.: strategies for solving sudoku or logical puzzles grids) as well as performed activities such as:

- Card playing (during the first three months),
- Use of cognitive software: “A gym for the mind”^1^ (the last three months),
- Exposure to a structured group discussion on topics encompassing relevant political and cultural events as well as a guided critique of the books read during the cognitive training,
- Preparation of sets of future cognitive activities for the group.

**Combination training: evaluation of compliance**

Adherence to the combination training schedule was controlled with two methods. First subjects were asked to note in a journal the times that activities were initiated and completed. This journal was collected at the end of the six months and compliance was evaluated. Subjects who missed more than 90% of the scheduled activities were classified as non-compliant and excluded from further analysis. The second method involved meeting every two weeks where the subjects were asked if they were having difficulties in maintaining the activity schedule and encouraged to keep up the good work or to try harder to maintain the schedule, as appropriate.

1 Arianna Ferrari, Donata Gollin, Anna Peruzzi (2007) Una palestra per la mente Stimolazione cognitiva per l'invecchiamento cerebrale e le demenze. Erickson editions.
